# Association between Lipid Profile and Apolipoproteins with Risk of Diabetic Foot Ulcer: A Systematic Review and Meta-Analysis

**DOI:** 10.1155/2022/5450173

**Published:** 2022-08-10

**Authors:** Juan R. Ulloque-Badaracco, Melany D. Mosquera-Rojas, Enrique A Hernandez-Bustamante, Esteban A Alarcón-Braga, Ricardo R. Ulloque-Badaracco, Ali Al-kassab-Córdova, Percy Herrera-Añazco, Vicente A. Benites-Zapata, Adrian V. Hernandez

**Affiliations:** ^1^Escuela de Medicina, Universidad Peruana de Ciencias Aplicadas, Lima, Peru; ^2^Sociedad Científica de Estudiantes de Medicina de la Universidad Peruana de Ciencias Aplicadas, Lima, Peru; ^3^Sociedad Científica de Estudiantes de Medicina de la Universidad Nacional de Trujillo, Trujillo, Peru; ^4^Grupo Peruano de Investigación Epidemiológica, Unidad para la Generación y Síntesis de Evidencias en Salud, Universidad San Ignacio de Loyola, Lima, Peru; ^5^Universidad Científica del Sur, Lima, Peru; ^6^Universidad Privada San Juan Bautista, Lima, Peru; ^7^Instituto de Evaluación de Tecnologías en Salud e Investigación—IETSI, EsSalud, Lima, Peru; ^8^Unidad de Investigación para la Generación y Síntesis de Evidencias en Salud, Vicerrectorado de Investigación, Universidad San Ignacio de Loyola, Lima, Peru; ^9^Unidad de Revisiones Sistemáticas y Meta-Análisis, Guías de Práctica Clínica y Evaluaciones de Tecnología Sanitaria, Vicerrectorado de Investigación, Universidad San Ignacio de Loyola, Lima, Peru; ^10^Health Outcomes, Policy, and Evidence Synthesis (HOPES) Group, University of Connecticut School of Pharmacy, Mansfield, CT, USA

## Abstract

**Background and Aims:**

Biomarkers are necessary to stratify the risk of diabetic foot ulcers (DFUs). This systematic review and meta-analysis aimed to evaluate the association between the lipid profile and apolipoproteins with the risk of DFU.

**Methods:**

A systematic search was conducted in PubMed, Scopus, Cochrane Library, and Web of Science among adult patients. Cohort and case-control studies were included. Random-effects models were used for meta-analyses, and the effects were expressed as odds ratio (OR) and their 95% confidence intervals (CIs). We evaluated publication bias through Egger's test and funnel plot.

**Results:**

A total of 12 cohort studies and 26 case-control studies were included, with 17076 patients. We found that the higher values of total cholesterol (TC), low-density lipoprotein (LDL), triglycerides, and lipoprotein(a) (Lp(a)) were associated with a higher risk of developing DFU (OR: 1.47, OR: 1.47, OR: 1.5, OR: 1.85, respectively). Otherwise, the lower values of HDL were associated with a higher risk of developing DFU (OR: 0.49). Publication bias was not found for associations between TC, HDL, LDL, or TG and the risk of DFU.

**Conclusions:**

The high values of LDL, TC, TG, and Lp(a) and low values of HDL are associated with a higher risk of developing DFU. Furthermore, we did not find a significant association for VLDL, ApoA1, ApoB, and ApoB/ApoA1 ratio.

## 1. Introduction

In 2019, the global prevalence of diabetes was estimated at 9.3%, which translates to 463 million people affected by this disease. It is expected that by 2030, the prevalence will increase to 10.2% and, by 2045, to 10.9% [1]. Among the most important complications of diabetes, the diabetic foot ulcer (DFU) emerges as a growing problem for public health systems since it is a leading cause of hospitalization and amputation in patients with diabetes [2]. Globally, the prevalence of DFU is estimated at 6.3%, and the highest prevalence has been found in North America (13.0%) [3]. Hence, adequate measures are necessary to tackle these data of concern.

Three factors are usually involved in DFU formation: diabetic neuropathy, trauma with secondary infection, and arterial occlusive disease [4]. Patients with diabetes often have an altered lipid profile and apolipoproteins, which contributes to the appearance of these factors [5]. These alterations are multifactorial; nevertheless, one of the most important alterations involves protein glycosylation, such as low-density lipoprotein (LDL). This event impedes the recognition of lipoproteins by cell receptors, and as a result, they remain in circulation for a more extended period [6]. Later, lipoproteins would be phagocytized by macrophages, and the synthesis of cholesterol esters will thus increase. When lipoproteins accumulate, they cause the transformation of macrophages into foam cells, which will form the fatty streak and, consequently, initiate the atherosclerotic process [7].

The most feared consequence of patients with DFU is amputation, which occurs in 6 to 43% of cases [8]. Currently, health care aimed at the care of DFU represents an average cost of $ 8,659 per person [9]. However, this pathology is preventable through certain measures such as lifestyle modification and lipid management [10]. Additionally, it is necessary to have tools to identify diabetic patients at higher risk of developing this pathology early, to prioritize surveillance. Biomarkers are valuable tools for these purposes. To date, multiple biomarkers focused on stratifying the risk of developing DFU have been studied, such as creatinine, lipid profile, apolipoproteins, hemoglobin A1c, platelet-to-lymphocyte ratio, and others [11, 12]. The lipid profile and apolipoproteins have been associated with complications in cardiovascular and neuropathic diseases, the same ones associated with the development of DFU; given this, several studies have evaluated their association [13]. Although a systematic review was published in 2014, it only included four studies [14], so an update is needed. Therefore, we aim to systematize the evidence regarding the association between the lipid profile and apolipoproteins with the risk of DFU.

## 2. Methods

This systematic review was registered in the International Prospective Register of Systematic Reviews (PROSPERO) (CD42022308926). In addition, we followed the Preferred Reporting Items for Systematic Reviews and Meta-analysis (PRISMA) statement [15] (see PRISMA checklist in Supplementary Table S1) and the Cochrane Handbook of Systematic Reviews [16].

### 2.1. Data Source and Search Strategy

A systematic search on articles evaluating the association between lipid biomarkers and apolipoproteins for the risk of developing DFU was carried out on December 20, 2021, in the following databases: PubMed, Scopus, Cochrane Library, and Web of Science. The search strategy was originally built in PubMed and adapted to the other databases (see Search Strategy in Supplementary Appendix 1). Additionally, we performed a manual search on preprint databases (Research Square and medRxiv) and other databases (China National Knowledge Infrastructure, Wangfang Data, VIP Chinese Science Journals Database, and CINAHL). The Peer Review of Electronic Search Strategies (PRESS) checklist was used to develop the search strategy. No language restrictions were applied. The biomarkers included in this systematic review are the following: total cholesterol (TC), LDL, high-density lipoprotein (HDL), triglycerides (TGs), very-low-density lipoprotein (VLDL), apolipoprotein B (ApoB), apolipoprotein A1 (ApoA1), ApoB/ApoA1 ratio, and lipoprotein(a) (Lp(a)). These markers were selected due to the lipid profile, as it is mainly comprised of them. Also, these were found in the systematic search of the available evidence for our research question.

### 2.2. Study Selection and Data Extraction

We included studies that met the following criteria: (1) evaluation of the association between lipid profile and the risk of developing a DFU, (2) case-control and cohort studies, and (3) adult patients (≥18 years). We excluded studies that were (1) case reports, (2) studies carried out in animals, (3) cross-sectional studies, (4) scoping reviews, (5) narrative reviews, (6) systematic reviews, (7) conference abstracts, and (8) letters to editors.

Regarding the selection process, after applying the search strategy to each database, the results were exported to the Rayyan QCRI program [17]. After eliminating duplicate studies, four authors independently analyzed the titles and abstracts of each article (RRU-B, MDM-R, EAA-B, and EAH-B). After identifying the potential literature to be included in the review, two reviewers analyzed the full text of each study independently (VAB-Z and PH-A) and verified that these met the selection criteria in their entirety. After this process, the articles were pooled, and duplicate studies were eliminated. In case of missing information, the authors of the articles were contacted. Finally, a secondary bibliographic search was carried out from the articles read in full text.

There was consensus among the authors in case of discrepancies about the inclusion/exclusion of an article at any stage of the selection process. We used a data extraction sheet built in Microsoft Excel for the data extraction. The following information was extracted from the selected articles independently by four authors (JRU-B, MDM-R, EAA-B, and EAH-B): number of participants, year of publication, study design, population characteristics, and association or exposure measures.

### 2.3. Evaluation of Study Quality and Publication Bias

Quality assessment was evaluated with the Newcastle-Ottawa Scale (NOS) [18] independently by two authors (AA-C and AVH). The maximum score was nine stars, and scores greater or equal to 6 were considered studies with low risk of bias (high quality), while studies with less than six stars were considered high risk of bias (low quality).

The publication bias assessment was evaluated through funnel plots and the Egger test [19]. A *p* value >0.1 was considered as indicative of no publication bias.

### 2.4. Data Synthesis and Analysis

The statistical analysis was performed using Review Manager 5.4 (RevMan 5.4) (The Cochrane Collaboration, Copenhagen, Denmark) and STATA Release 17.0 (College Station, TX: StataCorp LLC). Odds ratio (OR) and corresponding 95% conﬁdence intervals (CIs) were the only effective measures used. Values expressed as medians and their interquartile ranges (IQRs) were transformed into means and their corresponding standard deviations (SDs) using Hozo's method [20]. Chinn's method was employed to transform standard mean differences (SMDs) to their corresponding natural logarithm of the OR (ln(OR)) and its standard error [21]. The primary outcome analyzed was the risk of developing DFU.

We performed a random-effects meta-analysis of the reported OR in all cases. The heterogeneity of the selected studies was analyzed using the I^2^ statistic and Cochran's Q statistic. For the I^2^ statistic, *p* values ≥60% were considered a sign of severe heterogeneity, and for the Cochran's *Q* test, *p* values <0.1 were considered a sign of heterogeneity. Subgroup analyses were carried out by study location. Sensitivity analyses were performed using only studies with a low risk of bias. A *p* value <0.05 was considered statistically significant.

## 3. Results

### 3.1. Study Characteristics

**Figure 1:**
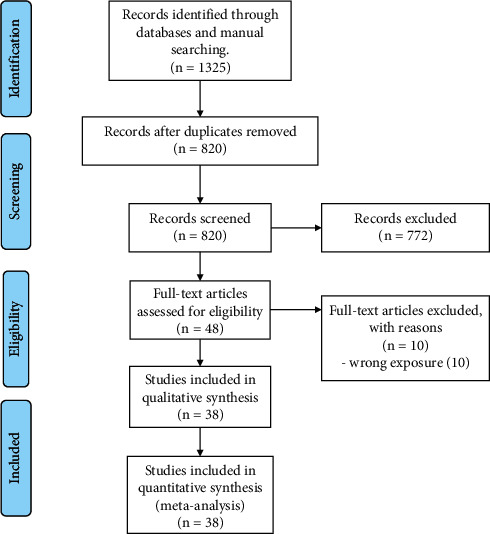
PRISMA flow diagram.

The systematic search yielded 1325 records, and 820 duplicates were removed. According to the eligibility criteria, after excluding articles by title and abstract and assessing their full-text documents, 38 were identified as eligible for this systematic review [22–59]. A PRISMA flow diagram summarizes the study selection process (Figure 1).

### 3.2. Study Characteristics

Characteristics from all included studies are summarized in Table 1. A total of 12 cohort studies and 26 case-control studies were included, wherein the relationship between TC, HDL, LDL, TG, VLDL, ApoA1, ApoB, ApoB/ApoA1 ratio, or Lp(a) and the risk of DFU was assessed. In addition, studies were conducted in China (10), Turkey (6), Japan (1), United Arab Emirates (1), Indonesia (2), India (6), Spain (1), Italy (2), Thailand (2), Oman (1), Sudan (1), Netherlands (1), Egypt (1), Greece (1), Scotland (1), and Iraq (1). The included studies were conducted between 2010 and 2021, accruing 17076 patients, of which 9418 were male, and 4019 developed DFU. The age range among all participants ranged from 20 to 88 years.

### 3.3. Evaluation of Study Quality

Regarding the evaluation of the quality of the studies with the NOS, 13 studies were at a low risk of bias (high quality), and the remaining 25 studies were at a high risk of bias (low quality) (Supplementary Table S2).

### 3.4. Association between TC and Risk of DFU

**Figure 2:**
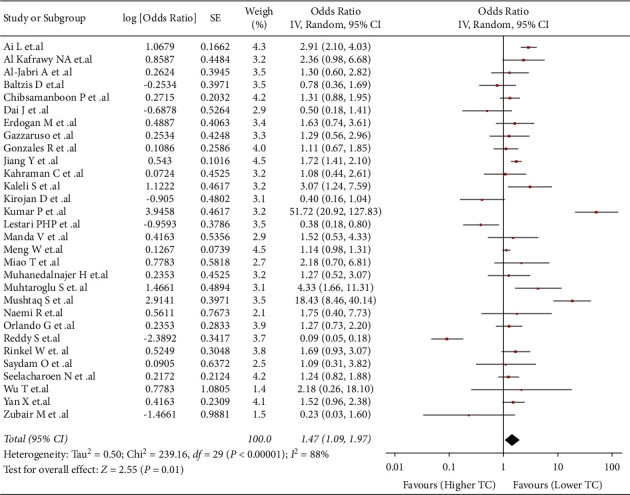
center34226500Association between TC and risk of DFU.

This association was evaluated in 30 studies (*n* = 9951). We found that higher values of TC were associated with higher risk of developing DFU (OR: 1.47; 95% CI: 1.09 to 1.97; *p* < 0.05; I^2^ = 88%) (Figure 2). Due to the severe heterogeneity, a subgroup analysis by countries was performed (Supplementary Figure S1). The Turkish studies subgroup (OR: 1.95; 95% CI: 1.15 to 3.32; *p* 0.28; I^2^ 37%) and other countries subgroup (OR: 1.09; 95% CI: 0.89 to 1.34; *p* 0.38; I^2^ 44%) did not exhibit statistically significant associations. The Chinese studies subgroup (OR: 1.76; 95% CI: 1.21 to 2.56; *p* < 0.05; I^2^ 66%) and Indian studies subgroup (OR: 2.91; 1 95% CI: 0.12 to 35.99; *p* 0.61; I^2^ 98%); there was only a decrease in heterogeneity in the Chinese studies subgroup. Additionally, the sensitivity analysis showed a nonstatistically significant association but with reduced heterogeneity (OR: 1.27; 95% CI: 0.95 to 1.70; *p* 0.1; I^2^ 44%) (Supplementary Figure S2).

### 3.5. Association between HDL and Risk of DFU

**Figure 3:**
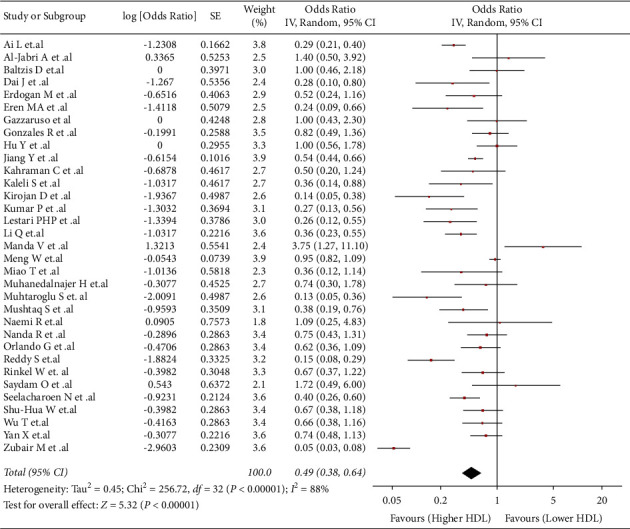
-32575531940500Association between HDL and risk of DFU.

This association was evaluated in 33 studies (*n* = 10982). We found that lower values of HDL were associated with a higher risk of developing DFU (OR: 0.49; 95% CI: 0.38 to 0.64; *p* < 0.05; I^2^ = 88%) (Figure 3). Due to the severe heterogeneity, a subgroup analysis by countries was performed (Supplementary Figure S3). All subgroups kept the statistically significant association, and just the Turkish studies subgroup did not show a decrease in heterogeneity. In sensitivity analysis, heterogeneity decreased, and the association remained (OR: 0.51; 95% CI: 0.39 to 0.67; *p* < 0.05; I^2^ = 37%) (Supplementary Figure S4).

### 3.6. Association between LDL and Risk of DFU

**Figure 4:**
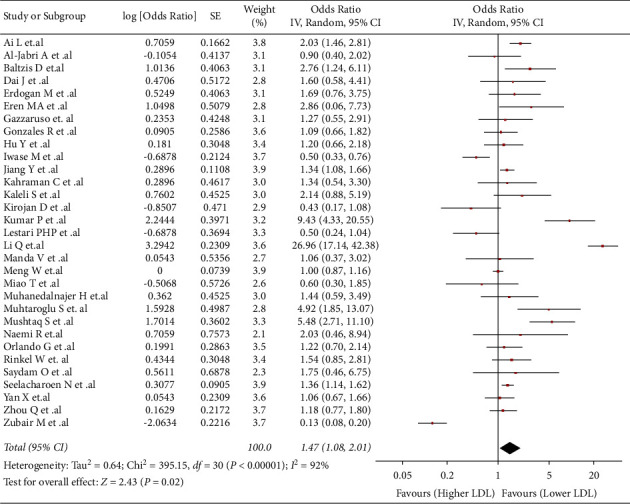
-43243528892500Association between LDL and risk of DFU.

This association was evaluated in 31 studies (*n* = 15570). We found that higher values of LDL were associated with a higher risk of developing DFU (OR: 1.47; 95% CI: 1.08 to 2.01; *p* < 0.05; I^2^ = 92%) (Figure 4). Due to the severe heterogeneity, a subgroup analysis by countries was performed (Supplementary Figure S5). The statistically significant association was lost in Chinese subgroup (OR: 1.96; 95% CI: 0.86 to 4.47; *p* = 0.11; I^2^ = 96%), Indian subgroup (OR: 1.89; 95% CI: 0.16 to 22.29; *p* = 0.61; I^2^ = 98%), and other countries subgroup (OR: 1.03; 95% CI: 0.82 to 1.29; *p* = 0.81; I^2^ = 67%). Turkish studies subgroup (OR: 2.17; 95% CI: 1.48 to 3.20; *p* < 0.001; I^2^ = 0%) was the only subgroup that kept the statistically significant association and showed a significant decrease in heterogeneity. The association was not statistically significant in sensitivity analysis but with low heterogeneity (OR: 1.25; 95% CI: 1.00 to 1.56; *p* = 0.05; I^2^ = 30%) (Supplementary Figure S6).

### 3.7. Association between TG and Risk of DFU

**Figure 5:**
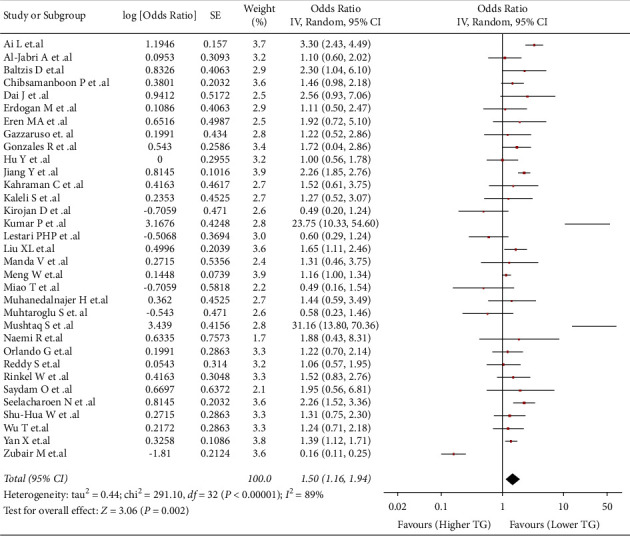
-58483522796500Association between TG and risk of DFU.

This association was evaluated in 33 studies (*n* = 11128). We found that higher values of TG were associated with a higher risk of developing DFU (OR: 1.5; 95% CI: 1.16 to 1.94; *p* < 0.05; I^2^ = 89%) (Figure 5). Due to the severe heterogeneity, a subgroup analysis by countries was performed (Supplementary Figure S7). The Chinese subgroup (OR 1.72; 95% CI: 1.26 to 2.33; *p* < 0.05; I^2^ = 79%) and other countries subgroup (OR: 1.3; 95% CI: 1.08 to 1.58; *p* < 0.05; I^2^ = 44%) were the only ones that kept the association and showed a decrease in heterogeneity. In sensitivity analysis, the association remained, and the heterogeneity decreased significantly (OR: 1.33; 95% CI: 1.03 to 1.73; *p* < 0.05; I^2^ = 39%) (Supplementary Figure S8).

### 3.8. Association between Lp(a) and Risk of DFU

This association was evaluated in two studies (*n* = 277). We found that higher values of Lp(a) were associated with a higher risk of developing DFU (OR: 1.85; 95% CI: 1.20 to 2.86; *p* < 0.05; I^2^ = 0%) (Supplementary Figure S9).

### 3.9. Association between ApoB and Risk of DFU

This association was evaluated in three studies (*n* = 870). However, no statistically significant association was found between ApoB and risk of DFU (OR: 2.48; 95% CI: 0.66 to 9.29; *p* = 0.18; I^2^ = 95%) (Supplementary Figure S10).

### 3.10. Association between ApoA1 and Risk of DFU

This association was evaluated in two studies (*n* = 422). However, no statistically significant association was found between ApoA1 and risk of DFU (OR: 0.01; 95% CI: 0.00 to 1151.83; *p* = 0.3; I^2^ = 100%) (Supplementary Figure S11).

### 3.11. Association between ApoB/ApoA1 Ratio and Risk of DFU

This association was evaluated in two studies (*n* = 422). However, no statistically significant association was found between ApoB/ApoA1 ratio and risk of DFU (OR: 3.08; 95% CI: 0.01 to 1214.41; *p* = 0.71; I^2^ = 99%) (Supplementary Figure S12).

### 3.12. Association between VLDL and Risk of DFU

This association was evaluated in three studies (*n* = 160). However, no statistically significant association was found between high VLDL values and risk of DFU (OR: 1.38; 95% CI: 0.74 to 2.57; *p* = 0.31; I^2^ = 0%) (Supplementary Figure S13).

### 3.13. Publication Bias

Publication bias was not found for associations between TC, HDL, LDL, or TG and the risk of DFU. The Egger test values were 0.73, 0.5, 0.46, and 0.86, respectively (Supplementary Figures S14, S15, S16, and S17).

## 4. Discussion

We found that a low level of HDL and a high level of TG and Lp(a) were associated with the development of DFU. However, there were significant regional variations regarding these associations. Most of the studies showed a high risk of bias. Moreover, in the sensitivity analysis, only the association between HDL and TG with DFU remained statistically significant, with consistently less heterogeneity.

Diabetic dyslipidemia (DD) is frequent, especially among patients with type 2 diabetes mellitus, where the prevalence is higher than 75% and is mainly mixed [60]. DD is linked to insulin resistance and can be an early manifestation prior to developing the diseased [61]. The main lipoprotein quantitative abnormality is the rise of TG. Meanwhile, the main qualitative abnormality of the DD is the increase in the sub-fraction of large VLDL and small and dense LDL particles [60].

DD is associated with a higher risk of cardiovascular events and peripheral vascular disease compared with subjects without diabetes, since it plays a central role in the genesis and progression of atherosclerosis [60–62]. Likewise, DD is associated with microvascular complications such as peripheral neuropathy related to the effect of fatty acids on mitochondrial traffic [63]. The deposition of these substances associated with lipid metabolism causes oxidative stress followed by increased expression of pro-inflammatory cytokines and neuronal apoptosis [64]. This neuropathic compromise is even associated with the initial lipid profile of the patient with a recent diagnosis of diabetes. Interestingly, an Anglo-Danish-Dutch study found that baseline waist circumference, body mass index, HDL, and LDL were associated with peripheral neuropathy 13 years after diabetes diagnosis [63].

These data are relevant to our research because both neuropathy and peripheral vascular disease are involved in the development of DFU [65]. In this sense, various studies found that a low level of HDL and a high level of TG were associated with an increase in diabetic peripheral neuropathy, while LDL did not show any association, which could explain our results [66, 67]. In addition, a study in diabetic patients in Iran found that serum Lp(a) level was positively correlated with the development of CVD, neuropathy, and diabetic nephropathy [68]. In particular, in patients with DFU, some studies showed that a low TG level was an independent risk factor for amputation of the affected limbo [69]. Similarly, another study showed that a low HDL level was associated with a lower incidence of lower limb amputation and death related to DFU [70]. This aspect is relevant because the participants evaluated in the studies included in our review include patients with some degree of ulceration, that is, with a higher risk of amputation.

A previously published systematic review only included four studies [14]. However, unlike our study, it did not include the evaluation of VLDL, Lp(a), or apolipoproteins. On the other hand, the search for articles was based on only two databases. Probably due to the small number of articles found, since the search lasted until September 2013, and the small sample in each of them, they did not perform a sensitivity analysis as in our case [14]. Although our results show that low HDL and high TG and Lp(a) levels can be used to predict the development of DFU, regional variations must be considered. There is a possibility that part of the explanation is due to variation in DD prevalence; for instance, DD prevalence is higher in the South Asian population [60]. Additionally, it is possible that regional variations in lipid profile could explain these differences. Although in general, the prevalence of total cholesterol is lower in Southwest Asian countries than in European and North or South American countries, their variations depend on the income level of the countries [71, 72]. Although in high-income countries the mean plasma cholesterol value decreased, in low- and middle-income countries it increased alongside with TG values [73]. Likewise, these values can vary between regions of large countries such as China [71]. Consequently, there are also regional variations in the use of statins for cholesterol management whose effects, in addition to lipid-lowering, could explain our findings. Indeed, statins are associated with cholesterol-independent effects by modulating the immune response, decreasing DD-associated oxidative stress, and stimulating fracture and wound healing [74]. In this sense, in addition to preventing macrovascular diseases, statins would also slow the progression of microvascular complications of diabetes by improving the ability of endothelial nitric oxide synthase to generate nitric oxide in endothelial cells regardless of lipid-lowering effects. Also, in addition to lowering lipid levels, statins can improve endothelial function and reduce oxidative stress, in turn, improving microvascular function [74, 75]. Thus, their use is increased in high-income countries [76], but in contrast, only a small percentage of patients in low-income countries have access to statins [77].

Although we have not evaluated the effect of nutritional status on our results, its influence on the lipid profile is well-known [78]. In this sense, as well as the regional variations of the lipid profile and the use of statins, it is possible that the regional variations of obesity and malnutrition explain the variations between countries in our findings [79].

However, aspects such as the possible effect of gender of the patients included in the studies have not been considered. For example, a study in Chinese diabetic patients showed that the ApoB/ApoA1 ratio predicted cardiovascular disease risk in men. In contrast, TC, LDL, ApoB, LDL/HDL, and TC/HDL were better predictors in women [80]. In addition, a systematic review found that there is a correlation between poor nutritional status and the presence of ulceration or delayed healing in patients with DFU [81]. Despite these biases, our results show the association of commonly used laboratory values with public health implications in this disease.

### 4.1. Limitations

Our study has several limitations. First, the studies did not adjust the values of the lipid markers with the confounding variables affecting the outcome under study. To avoid confounding bias, lifestyles, sociodemographic, and comorbidity variables should be considered and adjusted in future studies. Second, most of the studies have been carried out in the Asian continent, and very few have been found in other continents. Thus, it would be essential to analyze the value of these biomarkers in the risk of DFU in other latitudes. Third, high statistical heterogeneity was found due to the methodological and clinical differences between the studies. However, heterogeneity decreased when the sensitivity analysis was performed, which only included a low risk of bias studies. Finally, due to the lack of information in the studies, the values of sensitivity, specificity, and an optimal cutoff point of lipids for the risk of DFU were not estimated in a meta-analysis, so it should be addressed in future research.

## 5. Conclusions

High values of LDL, TC, TG, and Lp(a) and low values of HDL are associated with a higher risk of developing DFU. Furthermore, we did not find a statistically significant association for VLDL, ApoA1, ApoB, and ApoB/ApoA1 ratio. However, primary studies are needed to define the optimal cutoff point for these biomarkers according to the profile of different diabetic populations worldwide.

## Figures and Tables

**Table 1:** Characteristics of the included studies.

Author	Year	Country	Participants (male)	Mean/median age (SD/IQR)	Diabetes type of participants	Lipid marker analyzed	Lipid marker mean (SD) in diabetic patients with foot ulcer	Lipid marker mean (SD) in diabetic patients without foot ulcer
Iwase M et. al	2018	Japan	4870 (2755)	65.4 (10.2)	II	LDL	100.54 (32.09)	110.6 (26.29)

Zhou Q et. .al	2021	China	348 (348)	67.71 (8.35)	I and II	LDL	121.04 (28.61)	118.33 (29)

Manda V et. al	2012	United Arab Emirates	50 (NR)	NR	I and II	Total cholesterol	189.89 (66.79)	175.75 (56.49)
						VLDL	48.17 (41.29)	35.46 (16.31)
						HDL	37.28 (8.25)	31.34 (7.85)
						LDL	103.33 (39.31)	102.07 (39.61)
						Triglycerides	207.38 (122.24)	186.37 (149.3)

Lestari PHP et. al	2020	Indonesia	97 (45)	NR	II	Total cholesterol	170 (74)	204 (52)
						HDL	31 (19)	42 (9)
						LDL	112 (59)	132 (46)
						Triglycerides	142 (88)	169 (104)

Dai J et. al	2020	China	51 (29)	62 (5.93)	II	Total cholesterol	157.4 (54.14)	174.4 (34.8)
						HDL	34.8 (11.6)	42.62 (10.44)
						LDL	109.05 (31.71)	99.77 (37.51)
						Triglycerides	158.55 (87.69)	119.57 (61.12)

Mushtaq S et. al	2020	India	110 (NR)	NR	II	Total cholesterol	225.76 (38.19)	171.18 (28.21)
						HDL	39.27 (8)	42.94 (5.57)
						LDL	143.79 (44.82)	106.65 (32.63)
						Triglycerides	175.1 (27.74)	126.07 (23.2)

Jiang Y et. al	2015	China	1332 (784)	64 (55-72)	II	Total cholesterol	185.6 (45.8)	171.11 (48.7)
						HDL	42.53 (11.45)	46.4 (11.45)
						LDL	105.37 (37.24)	99.57 (37.22)
						Triglycerides	150.575 (91.85)	119.57 (52.48)

Ai L et. al	2020	China	1012 (551)	59.61 (12.15)	II	Total cholesterol	190.64 (46.4)	161.25 (50.66)
						HDL	34.8 (12.76)	42.15 (10.44)
						LDL	112.92 (32.87)	98.22 (38.67)
						Triglycerides	164.75 (135.52)	114.26 (60.23)

Kahraman C et. al	2014	Turkey	62 (33)	61.1 (11)	II	Total cholesterol	184.6 (43.8)	182.7 (43.4)
						HDL	38.8 (19.7)	45.1 (12.3)
						LDL	115.9 (39.1)	109.7 (36.8)
						Triglycerides	232.2 (153.8)	199.5 (126.2)

Eren MA et. al	2012	Turkey	54 (25)	56.2 (12.9)	II	HDL	30.9 (11.6)	38.67 (7.7)
						LDL	119.88 (34.8)	100.54 (30.9)
						Triglycerides	203.7 (141.72)	159.43 (97.43)

Erdogan M et. al	2010	Turkey	84 (36)	59.82 (10.55)	II	Total cholesterol	182.24 (39.34)	170.14 (51.08)
						HDL	36.12 (13.90)	40.97 (12.01)
						LDL	117.35 (34.77)	106.32 (41.65)
						Triglycerides	171 (89.11)	165.72 (92.74)

Gonzales R et. al	2010	Spain	198 (94)	68 (10.4)	II	Total cholesterol	201.1 (60.32)	198 (40.22)
						HDL	45.63 (12.37)	47.18 (15.08)
						ApoB	1.07 (0.3)	0.96 (0.25)
						LDL	132.64 (39.44)	130.32 (45.63)
						Lipoprotein (a)	1.43 (1.97)	1.02 (1.04)
						Triglycerides	163.86 (108.06)	133.75 (91.23)

Gazzaruso et. al	2011	Italy	79 (44)	57.2 (6.8)	II	Total cholesterol	204.95 (27.07)	201.1 (27.07)
						HDL	46.4 (7.734)	46.4 (7.734)
						Lipoprotein (a)	0.93 (0.81)	0.53 (0.69)
						LDL	127.6 (30.936)	123.74 (27.07)
						Triglycerides	168.3 (79.72)	159.43 (79.72)

Saydam O et. al	2021	Turkey	90 (18)	34 (26–52)	II	Total cholesterol	209.25 (43.7)	206.75 (46.6)
						HDL	31.75 (9.62)	29.25 (8.1)
						LDL	107.25 (34.81)	96.75 (33.3)
						Triglycerides	692.5 (522.96)	501.5 (509.62)

Zubair M et. al	2012	India	324 (205)	46.29 (13.19)	I and II	Total cholesterol	136.93 (13.7)	181.9 (32.3)
						HDL	34.6 (3.34)	44.3 (7.7)
						LDL	75.89 (18.34)	104.38 (30.1)
						Triglycerides	95.96 (21.7)	157.01 (83.1)

Hu Y et. al	2014	Saudi Arabia	598 (371)	53.2 (10.6)	II	HDL	46.4 (11.6)	46.4 (11.6)
						LDL	123.74 (42.54)	119.88 (38.67)
						Triglycerides	150.58 (141.72)	150.58 (106.29)

Miao T et. al	2020	China	53 (22)	54.2 (11.2)	II	Total cholesterol	180.97 (39.83)	167.83 (26.68)
						HDL	44.86 (6.574)	51.04 (11.98)
						LDL	100.93 (27.84)	109.05 (29.39)
						Triglycerides	128.43 (46.06)	172.72 (124.9)

Kalelí S et. al	2019	Turkey	64 (40)	NR	II	Total cholesterol	201.39 (30.21)	175.97 (48.23)
						HDL	36.23 (10.62)	43.36 (13.67)
						LDL	137.09 (29.94)	121.65 (41.22)
						Triglycerides	180.85 (61.06)	172.03 (71.48)

Kumar P et. al	2019	India	100 (48)	54.62 (10.84)	II	Total cholesterol	274.74 (7.6)	243.84 (18.4)
						HDL	27.34 (4.2)	31 (5.8)
						LDL	178.66 (4.08)	162.62 (17.67)
						Triglycerides	247.66 (21.98)	209.08 (21.7423)

Seelacharoen N et. al	2017	Thailand	300 (152)	61.23 (11.8)	II	Total cholesterol	201.29 (51.19)	195.83 (37.98)
						HDL	47.19 (12.84)	53.17 (10.18)
						LDL	121.28 (41.92)	114.85 (31.09)
						Triglycerides	183.06 (118.59)	139.72 (63.18)

Orlando G et. al	2021	Italy	175 (102)	72.6 (9.5)	I and II	Total cholesterol	181.5 (49.3)	175.7 (43.6)
						HDL	44.8 (14)	48 (10.9)
						LDL	106.1 (43.9)	101.4 (39.3)

Al-Jabri A et. al	2021	Oman	300 (137)	NR	II	Total cholesterol	NR	NR
						HDL	NR	NR
						LDL	NR	NR
						Triglycerides	NR	NR

Almobarak A et. al	2017	Sudan	310 (182)	58.7 (NR)	II	Total cholesterol	NR	NR
						HDL	NR	NR
						LDL	NR	NR
						Triglycerides	NR	NR

Rinkel W et. al	2021	Netherlands	410 (241)	65.7 (54.5–75.4)	I and II	Total cholesterol	168.21 (34.3)	157.5 (37.2)
						HDL	51.1 (14.2)	56 (22.8)
						ApoB	0.9 (0.44)	0.925 (0.22)
						LDL	80.23 (42.9)	72.49 (31.5)
						Triglycerides	168.28 (131.2)	146.1 (91.8)

Muhtaroglu S et. al	2015	Turkey	60 (32)	58.67 (9.23)	II	Total cholesterol	203.2 (28.8)	180.5 (26.6)
						HDL	33.37 (8.47)	42.93 (8.6)
						LDL	137.8 (34)	112.1 (22.9)
						Triglycerides	139.5 (69.6)	162.5 (80)

Chibsamanboon P et. al	2010	Thailand	364 (254)	NR	II	Total cholesterol	211.85 (56.30)	204.39 (45.69)
						Triglycerides	199.08 (128.19)	177.51 (89.69)

Kirojan D et. al	2017	Indonesia	60 (33)	NR	II	Total cholesterol	127.77 (35.73)	146 (36.04)
						HDL	20.47 (8.48)	32.33 (12.95)
						LDL	76.67 (28.71)	89.8 (29.15)
						Triglycerides	121.16 (65.31)	150.43 (83)

Wu T et. al	2021	China	172 (94)	55.1 (11.9)	II	Total cholesterol	177.88 (27.07)	189.5 (27.07)
						HDL	34.8 (19.3)	38.67 (11.6)
						Triglycerides	159.49 (70.86)	150.58 (79.72)

Reddy S et. al	2021	India	138 (87)	60.6 (10.1)	II	Total cholesterol	130.7 (40)	185 (42)
						HDL	32 (11)	44 (12)
						Triglycerides	147.5 (70.5)	145.5 (73)

Li Q et. al	2017	China	523 (281)	55.4 (9.8)	II	HDL	42.15 (10.8)	47.56 (8.89)
						LDL	206.1 (45.63)	136.5 (36.35)

Nanda R et. al	2021	India	160 (114)	52.7 (9.48)	II	HDL	33.15 (0.84)	41.46 (0.73)
						ApoA1	0.74 (0.031)	0.99 (0.026)

Liu XL et. al	2017	China	640 (NR)	NR	II	Triglycerides	NR	NR

Al Kafrawy NA et. al	2013	Egypt	100 (49)	57.4 (11.6)	I and II	Total cholesterol	NR	NR
Baltzis D et. al	2018	Greece	90 (55)	66 (9.2)	II	Total cholesterol	176.6 (44.1)	181.8 (34.4)
						HDL	44 (1.7)	44 (1.2)
						LDL	105 (5.3)	102.5 (3.9)
						Triglycerides	121 (10.3)	116.5 (9.5)

Naemi R et. al	2017	India	40 (30)	64.1 (9.4)	II	Total cholesterol	154 (27.3)	145.1 (28.8)
						HDL	41.3 (16.2)	40.8 (8.8)
						VLDL	25 (7)	22 (33)
						LDL	91.9 (31.3)	80.9 (26.7)
						Triglycerides	129.1 (34.6)	116.2 (36.9)

Meng W et. al	2017	Scotland	3394 (1916)	68.7 (9.06)	I and II	Total cholesterol	169 (32.48)	166.67 (31.71)
						HDL	52.2 (12.76)	52.59 (13.148)
						LDL	80.05 (24.36)	77.34 (23.2)
						Triglycerides	202.83 (117.8)	193.98 (111.6)

Yan X et. al	2021	China	262 (148)	55.3 (10.5)	II	Total cholesterol	182.13 (29)	175.95 (25.52)
						HDL	40.6 (10.44)	42.54 (11.988)
						LDL	110.98 (31.32)	110.2 (28.23)
						Triglycerides	84.3 (40.6)	77.73 (30.55)
						ApoB	1.13 (0.11)	0.81 (0.36)
						ApoA1	1.24 (0.22)	1.28 (0.18)
						ApoB/ApoA1 ratio	0.65 (0.28)	0.89 (0.16)

Muhanedalnajer H et. al	2020	Iraq	70 (45)	58 (8.87)	II	Total cholesterol	182.72 (52.99)	176.12 (44.52)
						HDL	32.61 (10.94)	34.58 (11.99)
						LDL	101 (51.14)	97.41 (50.23)
						Triglycerides	184.68 (76.10)	170.06 (65.22)
						VLDL	36.14 (15.26)	35.81 (14.03)

Shu-Hua W et. al	2021	China	160 (101)	55.05 (7.12)	II	HDL	44.47 (5.027)	45.63 (5.414)
						Triglycerides	201.06 (29.23)	195.75 (39.86)
						ApoB/ApoA1 ratio	0.93 (0.16)	0.62 (0.10)

NR: not reported; SMD: standardized mean difference; SD: standard deviation; OR: interquartile range.

## Data Availability

The data that support the findings of this study are available from the corresponding author upon request.

## Supplementary Materials

Supplementary Materials Appendix 1: Search strategy. Table S1. PRISMA checklist. Table S2. Newcastle-Ottawa Quality Assessment scale for included studies. Figure S1. Subgroup analysis according to the countries of the association between TC and risk of DFU. Figure S2. Sensitivity analysis according to the risk of bias of the association between TC and risk of DFU. Figure S3. Subgroup analysis according to countries of the association between HDL and risk of DFU. Figure S4. Sensitivity analysis according to the risk of bias of the association between HDL and risk of DFU. Figure S5. Subgroup analysis according to countries of the association between LDL and risk of DFU. Figure S6. Sensitivity analysis according to the risk of bias of the association between LDL and risk of DFU. Figure S7. Subgroup analysis according to countries of the association between TG and risk of DFU. Figure S8. Sensitivity analysis according to the risk of bias of the association between TG and risk of DFU. Figure S9. Association between Lp(a) and risk of DFU. Figure S10. Association between ApoB and risk of DFU. Figure S11. Association between ApoA1 and risk of DFU. Figure S12. Association between ApoB/ApoA1 ratio and risk of DFU. Figure S13. Association between VLDL and risk of DFU. Figure S14. A funnel plot of the studies that evaluated the association between TC and risk of DFU. Figure S14. B Egger test of all the studies that evaluated the association between TC and risk of DFU. Figure S15. A Funnel plot of the studies that evaluated the association between HDL and risk of DFU. Figure S15. B Egger test of all the studies that evaluated the association between HDL and risk of DFU. Figure S16. A Funnel plot of the studies that evaluated the association between LDL and risk of DFU. Figure S16. B Egger test of all the studies that evaluated the association between LDL and risk of DFU. Figure S17. A Funnel plot of the studies that evaluated the association between TG and risk of DFU. Figure S17. B Egger test of all the studies that evaluated the association between TG and risk of DFU. .
