# Supplementary material for: Association between Lipid Profile and Apolipoproteins with Risk of Diabetic Foot Ulcer: A Systematic Review and Meta-Analysis
Source: Int J Clin Pract. 2022 Aug 10;2022:5450173. doi: 10.1155/2022/5450173 (PMC9385316; doi:10.1155/2022/5450173)

**Association between lipid profile and apolipoproteins with risk of diabetic foot ulcer: A systematic review and meta-analysis**

**SUPPLEMENTARY MATERIAL**

**APPENDIX 1 :**  SEARCH STRATEGY

**PUBMED**

**Diabetes (#1)**

Diabetes Mellitus [MH] OR Diet, Diabetic [MH] OR Prediabetic State [MH] OR Glycation End Products, Advanced [MH] OR Glucose Intolerance [MH] OR “Diabetes Mellitus” [TIAB] OR “Prediabetic State” [TIAB] OR “Glucose Intolerance” [TIAB] OR “Diabetes Mellitus” [OT] OR “Prediabetic State” [OT] OR “Glucose Intolerance” [OT]

**Diabetic foot ulcer (#2)**

Diabetic Foot [MH] OR Foot, Diabetic [MH] OR Diabetic Feet [MH] OR Feet, Diabetic [MH] OR Foot Ulcer, Diabetic [MH] OR “Diabetic Foot” [TIAB] OR “Diabetic Feet” [TIAB] OR “Diabetic Foot” [OT] OR “Diabetic Feet” [OT] OR “Diabetic Foot Ulcer*” [TIAB] OR “Diabetic Foot Ulcer*” [OT]

**Triglycerides (#3)**

Triglycerides [MH] OR Triacylglycerol [MH] OR Triacylglycerols [MH] OR Triglyceride [MH] OR Triglycerides [TIAB] OR Triacylglycerol [TIAB] OR Triacylglycerols [TIAB] OR Triglyceride [TIAB] OR Triglycerides [OT] OR Triacylglycerol [OT] OR Triacylglycerols [OT] OR Triglyceride [OT]

**High-Density Lipoprotein Cholesterol (#4)**

Cholesterol, HDL [MH] OR alpha-Lipoprotein Cholesterol [MH] OR Cholesterol, alpha-Lipoprotein [MH] OR alpha Lipoprotein Cholesterol [MH] OR HDL Cholesterol [MH] OR High Density Lipoprotein Cholesterol [MH] OR Cholesterol, HDL2 [MH] OR HDL2 Cholesterol [MH] OR HDL(2) Cholesterol [MH] OR Cholesterol, HDL3 [MH] OR HDL3 Cholesterol [MH] OR HDL(3) Cholesterol [MH] OR “alpha-Lipoprotein Cholesterol” [TIAB] OR “alpha Lipoprotein Cholesterol” [TIAB] OR “HDL Cholesterol” [TIAB] OR “High Density Lipoprotein Cholesterol” [TIAB] OR “HDL2 Cholesterol” [TIAB] OR “HDL(2) Cholesterol” [TIAB] OR “HDL3 Cholesterol” [TIAB] OR “HDL(3) Cholesterol” [TIAB] OR “alpha-Lipoprotein Cholesterol” [OT] OR “alpha Lipoprotein Cholesterol” [OT] OR “HDL Cholesterol” [OT] OR “High Density Lipoprotein Cholesterol” [OT] OR “HDL2 Cholesterol” [OT] OR “HDL(2) Cholesterol” [OT] OR “HDL3 Cholesterol” [OT] OR “HDL(3) Cholesterol” [OT]

**Low-Density Lipoprotein Cholesterol (#5)**

Cholesterol, LDL [MH] OR Low Density Lipoprotein Cholesterol [MH] OR beta-Lipoprotein Cholesterol [MH] OR Cholesterol, beta-Lipoprotein [MH] OR beta Lipoprotein Cholesterol [MH] OR LDL Cholesterol [MH] OR Cholesteryl Linoleate, LDL [MH] OR LDL Cholesteryl Linoleate [MH] OR “Low Density Lipoprotein Cholesterol” [TIAB] OR “beta-Lipoprotein Cholesterol [TIAB] OR “beta Lipoprotein Cholesterol” [TIAB] OR “LDL Cholesterol” [TIAB] OR “LDL Cholesteryl Linoleate” [TIAB] OR “Low Density Lipoprotein Cholesterol” [OT] OR “beta-Lipoprotein Cholesterol [OT] OR “beta Lipoprotein Cholesterol” [OT] OR “LDL Cholesterol” [OT] OR “LDL Cholesteryl Linoleate” [OT]

**Cholesterol (#6)**

Cholesterol [MH] OR Epicholesterol [MH] OR Cholesterol [TIAB] OR Epicholesterol [TIAB] OR Cholesterol [OT] OR Epicholesterol [OT]

**Lipids (#7)**

Lipids [MH] OR Lipid [MH] OR Lipid* [TIAB] OR Lipid* [OT]

**Very Low Density Lipoprotein Cholesterol (#8)**

Cholesterol, VLDL [MH] OR VLDL Cholesterol [MH] OR Pre-beta-Lipoprotein Cholesterol [MH] OR Cholesterol, Pre-beta-Lipoprotein [MH] OR Pre beta Lipoprotein Cholesterol [MH] OR Very Low Density Lipoprotein Cholesterol [MH] OR Prebetalipoprotein Cholesterol [MH] OR Cholesterol, Prebetalipoprotein [MH] OR “VLDL Cholesterol” [TIAB] OR “Pre-beta-Lipoprotein Cholesterol” [TIAB] OR “Pre beta Lipoprotein Cholesterol” [TIAB] OR “Very Low Density Lipoprotein Cholesterol” [TIAB] OR “Prebetalipoprotein Cholesterol” [TIAB] OR “VLDL Cholesterol” [OT] OR “Pre-beta-Lipoprotein Cholesterol” [OT] OR “Pre beta Lipoprotein Cholesterol” [OT] OR “Very Low Density Lipoprotein Cholesterol” [OT] OR “Prebetalipoprotein Cholesterol” [OT]

**Formula**

(#1 AND #2) AND (#3 OR #4 OR #5 OR #6 OR #7 OR #8)

**SCOPUS**

(ALL(Diabetes Mellitus OR Diet, Diabetic OR Prediabetic State OR Glycation End Products, Advanced OR Glucose Intolerance OR Diabetes Mellitus OR Prediabetic State OR Glucose Intolerance OR Diabetes Mellitus OR Prediabetic State OR Glucose Intolerance) AND (ALL(Diabetic Foot OR Foot, Diabetic OR Diabetic Feet OR Feet, Diabetic OR Foot Ulcer, Diabetic OR Diabetic Foot OR Diabetic Feet OR Diabetic Foot OR Diabetic Feet OR Diabetic Foot Ulcer) AND ALL(Triglycerides OR Triacylglycerol OR Triacylglycerols ) OR ALL(HDL Cholesterol OR High Density Lipoprotein Cholesterol OR Cholesterol, HDL2 OR HDL2 Cholesterol OR HDL(2) Cholesterol OR Cholesterol, HDL OR alpha Lipoprotein Cholesterol OR HDL Cholesterol OR High Density Lipoprotein Cholesterol OR ALL(Cholesterol, LDL OR Low Density Lipoprotein Cholesterol OR beta-Lipoprotein Cholesterol OR Cholesterol, beta-Lipoprotein OR beta Lipoprotein Cholesterol OR LDL Cholesterol OR Cholesteryl Linoleate, LDL OR LDL Cholesteryl Linoleate OR Low Density Lipoprotein Cholesterol OR beta-Lipoprotein Cholesterol OR beta Lipoprotein Cholesterol OR LDL Cholesterol OR LDL Cholesteryl Linoleate OR Low Density Lipoprotein Cholesterol OR beta-Lipoprotein Cholesterol OR beta Lipoprotein Cholesterol OR LDL Cholesterol OR LDL Cholesteryl Linoleate) OR ALL(Cholesterol OR Epicholesterol OR Cholesterol OR Epicholesterol OR Cholesterol OR Epicholesterol) OR TITLE-ABS-KEY(Lipids OR Lipid) OR TITLE-ABS-KEY(Cholesterol, VLDL OR VLDL Cholesterol OR Pre-beta-Lipoprotein Cholesterol OR Cholesterol, Pre-beta-Lipoprotein OR Pre beta Lipoprotein Cholesterol OR Very Low Density Lipoprotein Cholesterol OR Prebetalipoprotein Cholesterol OR Cholesterol, Prebetalipoprotein OR VLDL Cholesterol OR Pre-beta-Lipoprotein Cholesterol OR Pre beta Lipoprotein Cholesterol OR Very Low Density Lipoprotein Cholesterol OR Prebetalipoprotein Cholesterol OR VLDL Cholesterol OR Pre-beta-Lipoprotein Cholesterol OR Pre beta Lipoprotein Cholesterol OR Very Low Density Lipoprotein Cholesterol OR Prebetalipoprotein Cholesterol))

**COCHRANE LIBRARY**

(Diabetes Mellitus OR Diet, Diabetic OR Prediabetic State OR Glycation End Products, Advanced OR Glucose Intolerance OR Diabetes Mellitus OR Prediabetic State OR Glucose Intolerance OR Diabetes Mellitus OR Prediabetic State OR Glucose Intolerance) AND (Diabetic Foot OR Foot, Diabetic OR Diabetic Feet OR Feet, Diabetic OR Foot Ulcer, Diabetic OR Diabetic Foot OR Diabetic Feet OR Diabetic Foot OR Diabetic Feet OR Diabetic Foot Ulcer):ti,ab,kw AND (Triglycerides OR Triacylglycerol OR Triacylglycerols OR Triglyceride OR Triglycerides OR Triacylglycerol OR Triacylglycerols OR Triglyceride OR Triglycerides OR Triacylglycerol OR Triacylglycerols OR Triglyceride) OR (Cholesterol, HDL OR alpha-Lipoprotein Cholesterol OR Cholesterol, alpha-Lipoprotein OR alpha Lipoprotein Cholesterol OR HDL Cholesterol OR High Density Lipoprotein Cholesterol OR Cholesterol, HDL2 OR HDL2 Cholesterol OR HDL(2) Cholesterol OR Cholesterol, HDL3 OR HDL3 Cholesterol OR HDL(3) Cholesterol OR alpha-Lipoprotein Cholesterol OR alpha Lipoprotein Cholesterol OR HDL Cholesterol OR High Density Lipoprotein Cholesterol OR HDL2 Cholesterol OR HDL(2) Cholesterol OR HDL3 Cholesterol OR HDL(3) Cholesterol OR alpha-Lipoprotein Cholesterol OR alpha Lipoprotein Cholesterol OR HDL Cholesterol OR High Density Lipoprotein Cholesterol OR HDL2 Cholesterol OR HDL(2) Cholesterol OR HDL3 Cholesterol OR HDL(3) Cholesterol):ti,ab,kw OR (Cholesterol, LDL OR Low Density Lipoprotein Cholesterol OR beta-Lipoprotein Cholesterol OR Cholesterol, beta-Lipoprotein OR beta Lipoprotein Cholesterol OR LDL Cholesterol OR Cholesteryl Linoleate, LDL OR LDL Cholesteryl Linoleate OR Low Density Lipoprotein Cholesterol OR beta-Lipoprotein Cholesterol OR beta Lipoprotein Cholesterol OR LDL Cholesterol OR LDL Cholesteryl Linoleate OR Low Density Lipoprotein Cholesterol OR beta-Lipoprotein Cholesterol OR beta Lipoprotein Cholesterol OR LDL Cholesterol OR LDL Cholesteryl Linoleate):ti,ab,kw OR (Cholesterol OR Epicholesterol OR Cholesterol OR Epicholesterol OR Cholesterol OR Epicholesterol):ti,ab,kw OR(Lipids OR Lipid):ti,ab,kw OR (Cholesterol, VLDL OR VLDL Cholesterol OR Pre-beta-Lipoprotein Cholesterol OR Cholesterol, Pre-beta-Lipoprotein OR Pre beta Lipoprotein Cholesterol OR Very Low Density Lipoprotein Cholesterol OR Prebetalipoprotein Cholesterol OR Cholesterol, Prebetalipoprotein OR VLDL Cholesterol OR Pre-beta-Lipoprotein Cholesterol OR Pre beta Lipoprotein Cholesterol OR Very Low Density Lipoprotein Cholesterol OR Prebetalipoprotein Cholesterol OR VLDL Cholesterol OR Pre-beta-Lipoprotein Cholesterol OR Pre beta Lipoprotein Cholesterol OR Very Low Density Lipoprotein Cholesterol OR Prebetalipoprotein Cholesterol):ti,ab,kw

**WEB OF SCIENCE**

((((((ALL=(Diabetes Mellitus )) AND ALL=(Diabetic Foot OR Foot, Diabetic OR Diabetic Feet OR Feet, Diabetic OR Foot Ulcer, Diabetic OR Diabetic Foot OR Diabetic Feet OR Diabetic Foot OR Diabetic Feet OR Diabetic Foot Ulcer* )) AND ALL=(Triglycerides OR Triacylglycerol OR Triacylglycerols OR Triglyceride OR Triglycerides OR Triacylglycerol OR Triacylglycerols OR Triglyceride OR Triglycerides OR Triacylglycerol OR Triacylglycerols OR Triglyceride )) OR ALL=(Cholesterol, HDL OR alpha-Lipoprotein Cholesterol OR Cholesterol, alpha-Lipoprotein OR alpha Lipoprotein Cholesterol OR HDL Cholesterol OR High Density Lipoprotein Cholesterol OR Cholesterol, HDL2 OR HDL2 Cholesterol OR HDL(2) Cholesterol OR Cholesterol, HDL3 OR HDL3 Cholesterol OR HDL(3) Cholesterol OR alpha-Lipoprotein Cholesterol OR alpha Lipoprotein Cholesterol OR HDL Cholesterol OR High Density Lipoprotein Cholesterol OR HDL2 Cholesterol OR HDL(2) Cholesterol OR HDL3 Cholesterol OR HDL(3) Cholesterol OR alpha-Lipoprotein Cholesterol OR alpha Lipoprotein Cholesterol OR HDL Cholesterol OR High Density Lipoprotein Cholesterol OR HDL2 Cholesterol OR HDL(2) Cholesterol OR HDL3 Cholesterol OR HDL(3) Cholesterol )) OR ALL=(Cholesterol, LDL OR Low Density Lipoprotein Cholesterol OR beta-Lipoprotein Cholesterol OR Cholesterol, beta-Lipoprotein OR beta Lipoprotein Cholesterol OR LDL Cholesterol OR Cholesteryl Linoleate, LDL OR LDL Cholesteryl Linoleate OR Low Density Lipoprotein Cholesterol OR beta-Lipoprotein Cholesterol OR beta Lipoprotein Cholesterol OR LDL Cholesterol OR LDL Cholesteryl Linoleate OR Low Density Lipoprotein Cholesterol OR beta-Lipoprotein Cholesterol OR beta Lipoprotein Cholesterol OR LDL Cholesterol OR LDL Cholesteryl Linoleate )) OR ALL=(Cholesterol, LDL OR Low Density Lipoprotein Cholesterol OR beta-Lipoprotein Cholesterol OR Cholesterol, beta-Lipoprotein OR beta Lipoprotein Cholesterol OR LDL Cholesterol OR Cholesteryl Linoleate, LDL OR LDL Cholesteryl Linoleate OR Low Density Lipoprotein Cholesterol OR beta-Lipoprotein Cholesterol OR beta Lipoprotein Cholesterol OR LDL Cholesterol OR LDL Cholesteryl Linoleate OR Low Density Lipoprotein Cholesterol OR beta-Lipoprotein Cholesterol OR beta Lipoprotein Cholesterol OR LDL Cholesterol OR LDL Cholesteryl Linoleate )) OR ALL=(Cholesterol, VLDL OR VLDL Cholesterol OR Pre-beta-Lipoprotein Cholesterol OR Cholesterol, Pre-beta-Lipoprotein OR Pre beta Lipoprotein Cholesterol OR Very Low Density Lipoprotein Cholesterol OR Prebetalipoprotein Cholesterol OR Cholesterol, Prebetalipoprotein OR VLDL Cholesterol OR Pre-beta-Lipoprotein Cholesterol OR Pre beta Lipoprotein Cholesterol OR Very Low Density Lipoprotein Cholesterol OR Prebetalipoprotein Cholesterol OR VLDL Cholesterol OR Pre-beta-Lipoprotein Cholesterol OR Pre beta Lipoprotein Cholesterol OR Very Low Density Lipoprotein Cholesterol OR Prebetalipoprotein Cholesterol )

**Table S1. Prisma checklist**

| **Section and Topic** | **Item #** | **Checklist item** | **Location where item is reported** |
| --- | --- | --- | --- |
| **TITLE** | | |  |
| Title | 1 | Identify the report as a systematic review. | Title Page |
| **ABSTRACT** | | |  |
| Abstract | 2 | See the PRISMA 2020 for Abstracts checklist. | Title Page |
| **INTRODUCTION** | | |  |
| Rationale | 3 | Describe the rationale for the review in the context of existing knowledge. | Lines 17 -26 of the introduction section |
| Objectives | 4 | Provide an explicit statement of the objective(s) or question(s) the review addresses. | Lines 29 -30 of the introduction section |
| **METHODS** | | |  |
| Eligibility criteria | 5 | Specify the inclusion and exclusion criteria for the review and how studies were grouped for the syntheses. | Subheading 2.2 |
| Information sources | 6 | Specify all databases, registers, websites, organisations, reference lists and other sources searched or consulted to identify studies. Specify the date when each source was last searched or consulted. | Subheading 2.1 |
| Search strategy | 7 | Present the full search strategies for all databases, registers and websites, including any filters and limits used. | Subheading 2.1 |
| Selection process | 8 | Specify the methods used to decide whether a study met the inclusion criteria of the review, including how many reviewers screened each record and each report retrieved, whether they worked independently, and if applicable, details of automation tools used in the process. | Subheading 2.2 |
| Data collection process | 9 | Specify the methods used to collect data from reports, including how many reviewers collected data from each report, whether they worked independently, any processes for obtaining or confirming data from study investigators, and if applicable, details of automation tools used in the process. | Subheading 2.2 |
| Data items | 10a | List and define all outcomes for which data were sought. Specify whether all results that were compatible with each outcome domain in each study were sought (e.g. for all measures, time points, analyses), and if not, the methods used to decide which results to collect. | Subheadings 2.1 and 2.4 |
|  | 10b | List and define all other variables for which data were sought (e.g. participant and intervention characteristics, funding sources). Describe any assumptions made about any missing or unclear information. | Subheadings 2.1 and 2.4 |
| Study risk of bias assessment | 11 | Specify the methods used to assess risk of bias in the included studies, including details of the tool(s) used, how many reviewers assessed each study and whether they worked independently, and if applicable, details of automation tools used in the process. | Subheading 2.3 |
| Effect measures | 12 | Specify for each outcome the effect measure(s) (e.g. risk ratio, mean difference) used in the synthesis or presentation of results. | Subheading 2.4 |
| Synthesis methods | 13a | Describe the processes used to decide which studies were eligible for each synthesis (e.g. tabulating the study intervention characteristics and comparing against the planned groups for each synthesis (item #5)). | Subheading 2.4 |
|  | 13b | Describe any methods required to prepare the data for presentation or synthesis, such as handling of missing summary statistics, or data conversions. | Subheading 2.4 |
|  | 13c | Describe any methods used to tabulate or visually display results of individual studies and syntheses. | Subheading 2.4 |
|  | 13d | Describe any methods used to synthesize results and provide a rationale for the choice(s). If meta-analysis was performed, describe the model(s), method(s) to identify the presence and extent of statistical heterogeneity, and software package(s) used. | Subheading 2.4 |
|  | 13e | Describe any methods used to explore possible causes of heterogeneity among study results (e.g. subgroup analysis, meta-regression). | Subheading 2.4 |
|  | 13f | Describe any sensitivity analyses conducted to assess robustness of the synthesized results. | Subheading 2.4 |
| Reporting bias assessment | 14 | Describe any methods used to assess risk of bias due to missing results in a synthesis (arising from reporting biases). | Subheading 2.3 |
| Certainty assessment | 15 | Describe any methods used to assess certainty (or confidence) in the body of evidence for an outcome. | Subheading 2.4 |
| **RESULTS** | | |  |
| Study selection | 16a | Describe the results of the search and selection process, from the number of records identified in the search to the number of studies included in the review, ideally using a flow diagram. | Subheading 3.1 |
|  | 16b | Cite studies that might appear to meet the inclusion criteria, but which were excluded, and explain why they were excluded. | Subheading 3.1 |
| Study characteristics | 17 | Cite each included study and present its characteristics. | Subheading 3.1 |
| Risk of bias in studies | 18 | Present assessments of risk of bias for each included study. | Subheading 3.3 |
| Results of individual studies | 19 | For all outcomes, present, for each study: (a) summary statistics for each group (where appropriate) and (b) an effect estimate and its precision (e.g. confidence/credible interval), ideally using structured tables or plots. | Subheadings 3.4 – 3.12 |
| Results of syntheses | 20a | For each synthesis, briefly summarise the characteristics and risk of bias among contributing studies. | Subheading 3.2 |
|  | 20b | Present results of all statistical syntheses conducted. If meta-analysis was done, present for each the summary estimate and its precision (e.g. confidence/credible interval) and measures of statistical heterogeneity. If comparing groups, describe the direction of the effect. | Subheadings 3.4 – 3.12 |
|  | 20c | Present results of all investigations of possible causes of heterogeneity among study results. | Subheadings 3.4 – 3.12 |
|  | 20d | Present results of all sensitivity analyses conducted to assess the robustness of the synthesized results. | Subheadings 3.4 – 3.7 |
| Reporting biases | 21 | Present assessments of risk of bias due to missing results (arising from reporting biases) for each synthesis assessed. | Subheading 3.13 |
| Certainty of evidence | 22 | Present assessments of certainty (or confidence) in the body of evidence for each outcome assessed. | Subheadings 3.4 – 3.7 |
| **DISCUSSION** | | |  |
| Discussion | 23a | Provide a general interpretation of the results in the context of other evidence. | Lines 1 -65 of the discussion section |
|  | 23b | Discuss any limitations of the evidence included in the review. | Subheading 4.1 |
|  | 23c | Discuss any limitations of the review processes used. | Subheading 4.1 |
|  | 23d | Discuss implications of the results for practice, policy, and future research. | Lines 1 -65 of the discussion section |
| **OTHER INFORMATION** | | |  |
| Registration and protocol | 24a | Provide registration information for the review, including register name and registration number, or state that the review was not registered. | Lines 1 -4 of the Methods section |
|  | 24b | Indicate where the review protocol can be accessed, or state that a protocol was not prepared. | Lines 1 -4 of the Methods section |
|  | 24c | Describe and explain any amendments to information provided at registration or in the protocol. | Lines 1 -4 of the Methods section |
| Support | 25 | Describe sources of financial or non-financial support for the review, and the role of the funders or sponsors in the review. | End of manuscript before references |
| Competing interests | 26 | Declare any competing interests of review authors. | End of manuscript before references |
| Availability of data, code and other materials | 27 | Report which of the following are publicly available and where they can be found: template data collection forms; data extracted from included studies; data used for all analyses; analytic code; any other materials used in the review. | End of manuscript before references |

**Table S2.Newcastle - Ottawa Quality Assessment scale for included studies**

| **NEWCASTLE - OTTAWA QUALITY ASSESSMENT SCALE FOR COHORT STUDIES** | | | | | | | | | | |
| --- | --- | --- | --- | --- | --- | --- | --- | --- | --- | --- |
| **STUDY** | **SELECTION** | | | | **COMPARABILITY** | **OUTCOME** | | |  |  |
|  | **Representativeness of the exposed cohort** | **Selection of the non-exposed cohort** | **Ascertainment of exposure** | **Demonstration that outcome of interest was not present at start of study** | **Comparability of Cohorts on the Basis of the Design or Analysis Maximum : ☆☆** | **Assessment of outcome** | **Was follow-up long enough for outcomes to occur** | **Adequacy of follow up of cohorts** | **SCORE** | **Evidence quality** |
| Iwase M et .al | **☆** |  | **☆** |  | **☆** | **☆** |  | **☆** | *5* | High Risk of bias |
| Zhou Q et .al | **☆** |  | **☆** |  |  | **☆** | **☆** | **☆** | *5* | High Risk of bias |
| Saydam O et .al | **☆** | **☆** | **☆** | **☆** | **☆** | **☆** | **☆** | **☆** | *8* | Low Risk of Bias |
| Zubair M et .al | **☆** |  | **☆** |  | **☆** | **☆** |  | **☆** | *5* | High Risk of bias |
| Orlando G et .al | **☆** | **☆** | **☆** | **☆** | **☆** | **☆** | **☆** | **☆** | *8* | Low Risk of Bias |
| Jiang Y et .al | **☆** | **☆** |  | **☆** |  |  | **☆** | **☆** | *5* | High Risk of bias |
| Ai L et .al | **☆** |  | **☆** |  | **☆** |  | **☆** | **☆** | *5* | High Risk of bias |
| Rinkel W et. al | **☆** | **☆** | **☆** | **☆** | **☆☆** | **☆** | **☆** | **☆** | *9* | Low Risk of Bias |
| Chibsamanboon P et .al | **☆** |  | **☆** |  |  | **☆** | **☆** |  | *4* | High Risk of bias |
| Li Q et.al | **☆** |  | **☆** |  | **☆** | **☆** |  | **☆** | *5* | High Risk of bias |
| Liu XL et .al | **☆** |  | **☆** |  |  | **☆** | **☆** | **☆** | *5* | High Risk of bias |
| Naemi R et.al | **☆** | **☆** | **☆** |  |  |  | **☆** | **☆** | *5* | High Risk  of bias |

| **NEWCASTLE - OTTAWA QUALITY ASSESSMENT SCALE FOR CASE-CONTROL STUDIES** | | | | | | | | | | | | | |  |  |  |
| --- | --- | --- | --- | --- | --- | --- | --- | --- | --- | --- | --- | --- | --- | --- | --- | --- |
| **STUDY** | **SELECTION** | | | | | **COMPARABILITY** | | **OUTCOME** | | | |  | |  |  |  |
|  | | **Is the case definition adequate?** | **Representativeness of the cases** | **Selection of Controls** | **Definition of Controls** | **Comparability of cases and controls on the basis of the design or analysis (Maximum : ☆☆ )** | | **Ascertainment of exposure** | | **Same method of ascertainment for cases and controls** | **Non-Response rate** | **SCORE** | | | **Evidence**  **quality** | |
| Manda V et .al | | **☆** |  |  |  | **☆** | |  | | **☆** | **☆** | *4* | | | High Risk of bias | |
| Lestari PHP et .al | | **☆** | **☆** | **☆** | **☆** | **☆** | | **☆** | | **☆** | **☆** | *8* | | | Low Risk of Bias | |
| Dai J et .al | | **☆** | **☆** |  | **☆** |  | |  | | **☆** | **☆** | *5* | | | High Risk of bias | |
| Mushtaq S et .al | | **☆** | **☆** | **☆** |  |  | | **☆** | | **☆** |  | *5* | | | High Risk of bias | |
| Kirojan D et .al | | **☆** |  | **☆** |  | **☆** | |  | | **☆** | **☆** | *5* | | | High Risk of bias | |
| Kahraman C et .al | | **☆** | **☆** | **☆** |  | **☆** | | **☆** | | **☆** | **☆** | *7* | | | Low Risk of Bias | |
| Eren MA et .al | | **☆** | **☆** | **☆** | **☆** | **☆** | | **☆** | | **☆** | **☆** | *8* | | | Low Risk of Bias | |
| Erdogan M et .al | | **☆** |  | **☆** | **☆** | **☆** | |  | | **☆** | **☆** | *6* | | | Low Risk of Bias | |
| Gonzales R et .al | | **☆** | **☆** | **☆** |  | **☆** | | **☆** | | **☆** | **☆** | *7* | | | Low Risk of Bias | |
| Gazzaruso et. al | | **☆** | **☆** | **☆** | **☆** | **☆** | | **☆** | | **☆** | **☆** | *8* | | | Low Risk of Bias | |
| Hu Y et .al | | **☆** | **☆** |  | **☆** |  | | **☆** | |  |  | *4* | | | High Risk of bias | |
| Miao T et .al | | **☆** | **☆** | **☆** | **☆** | **☆** | | **☆** | | **☆** | **☆** | *8* | | | Low Risk of Bias | |
| Kalelí S et .al | | **☆** | **☆** | **☆** |  | **☆** | | **☆** | | **☆** | **☆** | *7* | | | Low Risk of Bias | |
| Kumar P et .al | | **☆** |  | **☆** |  |  | | **☆** | | **☆** | **☆** | *5* | | | High Risk of bias | |
| Seelacharoen N et .al | | **☆** | **☆** | **☆** | **☆** | **☆** | | **☆** | | **☆** | **☆** | *8* | | | Low Risk of Bias | |
| Al-Jabri A et .al | | **☆** |  | **☆** | **☆** | **☆** | | **☆** | | **☆** | **☆** | *7* | | | Low Risk of Bias | |
| Muhtaroglu S et. al | | **☆** |  | **☆** |  |  | | **☆** | | **☆** | **☆** | *5* | | | High Risk of bias | |
| Wu T et.al | | **☆** |  | **☆** |  |  | | **☆** | | **☆** | **☆** | *5* | | | High Risk of bias | |
| Reddy S et.al | | **☆** |  | **☆** |  | **☆** | | **☆** | |  | **☆** | *5* | | | High Risk of bias | |
| Nanda R et .al | | **☆** |  | **☆** |  |  | | **☆** | | **☆** | **☆** | *5* | | | High Risk of bias | |
| Baltzis D et.al | | **☆** |  | **☆** |  |  | | **☆** | | **☆** |  | *4* | | | High Risk of bias | |
| Al Kafrawy NA et.al | | **☆** |  | **☆** |  | **☆** | |  | | **☆** | **☆** | *5* | | | High Risk of bias | |
| Meng W et.al | | **☆** | **☆** |  |  | **☆** | |  | | **☆** | **☆** | *5* | | | High Risk of bias | |
| Yan X et .al | | **☆** |  | **☆** |  | **☆** | |  | | **☆** | **☆** | *5* | | | High Risk of bias | |
| Muhanedalnajer H et.al | | **☆** |  | **☆** |  | **☆** | |  | | **☆** | **☆** | *5* | | | High Risk of bias | |
| Shu-Hua W et.al | | **☆** |  | **☆** |  | **☆** | |  | | **☆** |  | *4* | | | High Risk of bias | |

**Figure S1. Subgroup analysis according to the countries of the association between TC and risk of DFU.**

**
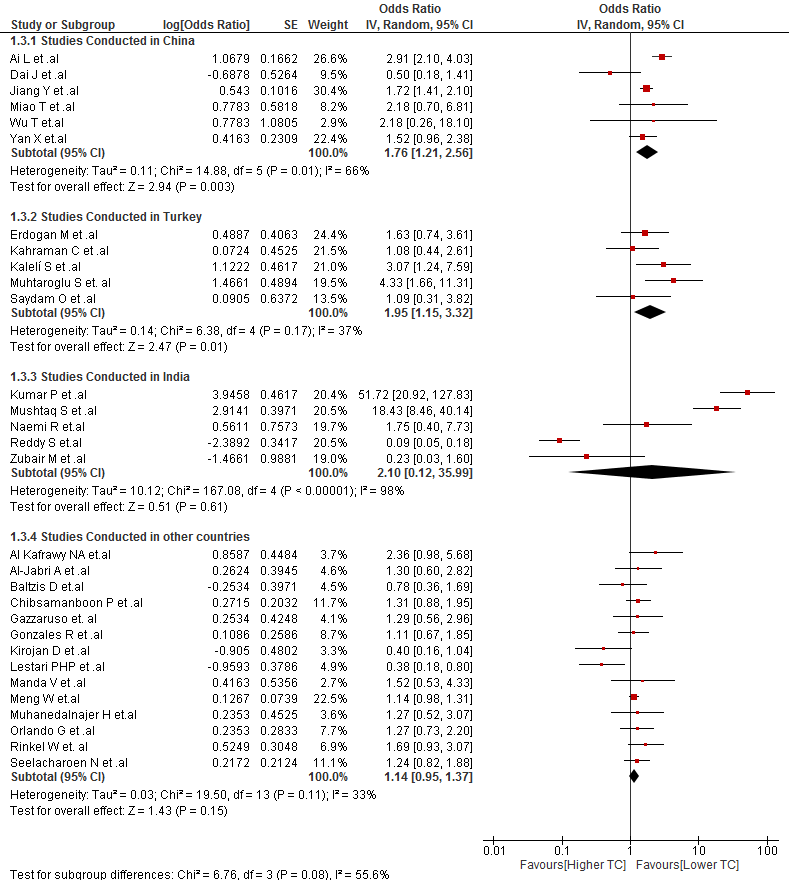
**

**Figure S2. Sensitivity analysis according to risk of bias of the association between TC and risk of DFU**

**
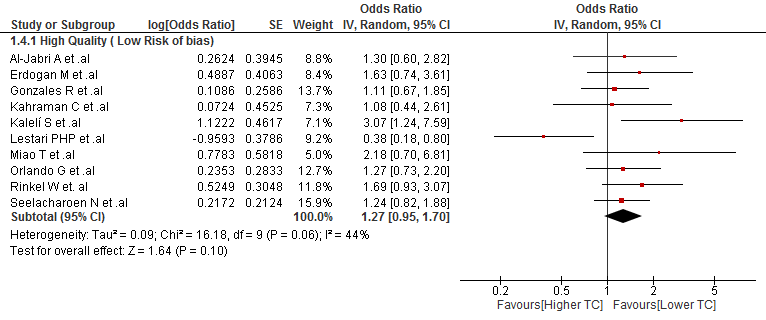
**

**Figure S3. Subgroup analysis according to countries of the association between HDL and risk of DFU**

**
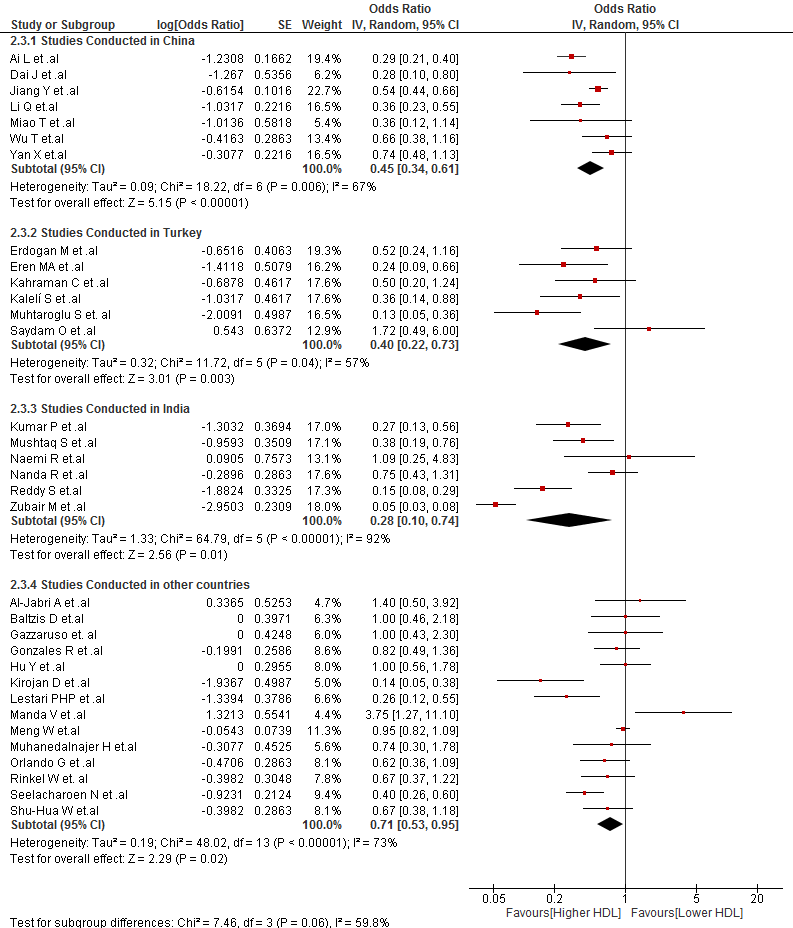
**

**Figure S4. Sensitivity analysis according to risk of bias of the association between HDL and risk of DFU.**

**
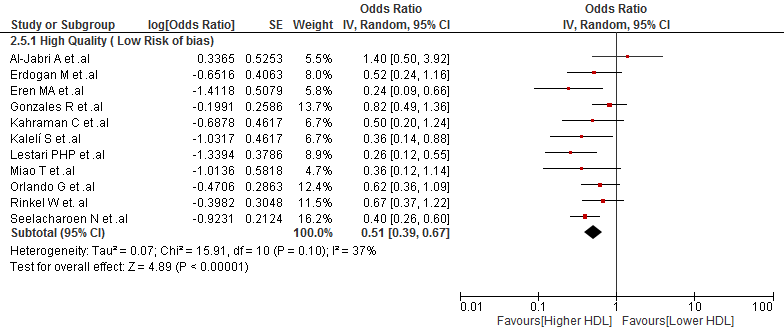
**

**Figure S5. Subgroup analysis according to countries of the association between LDL and risk of DFU**

**
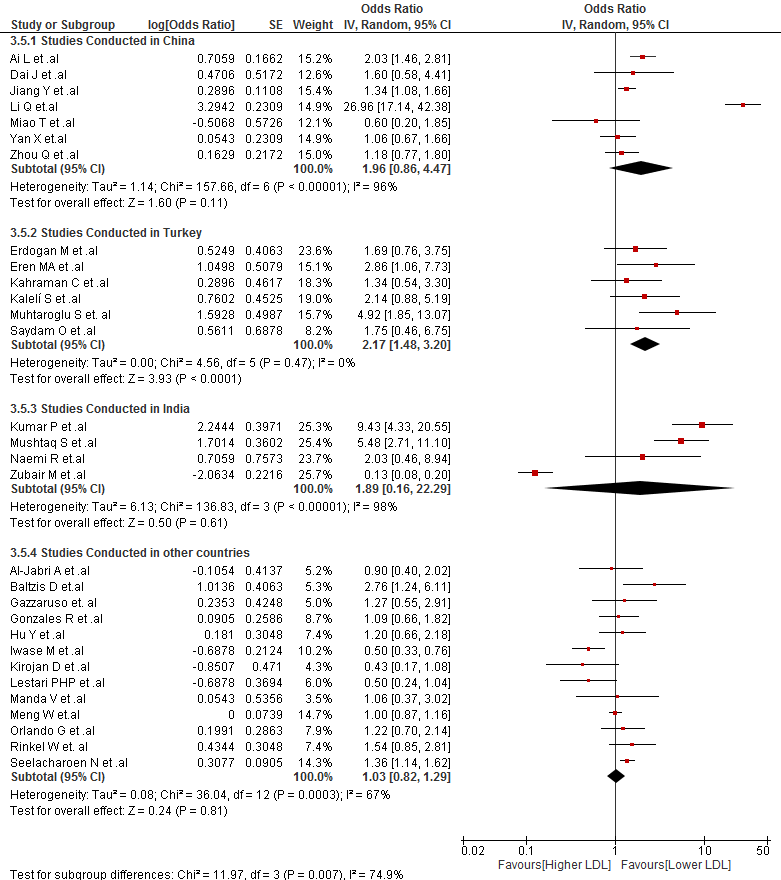
**

**Figure S6. Sensitivity analysis according to risk of bias of the association between LDL and risk of DFU.**

**
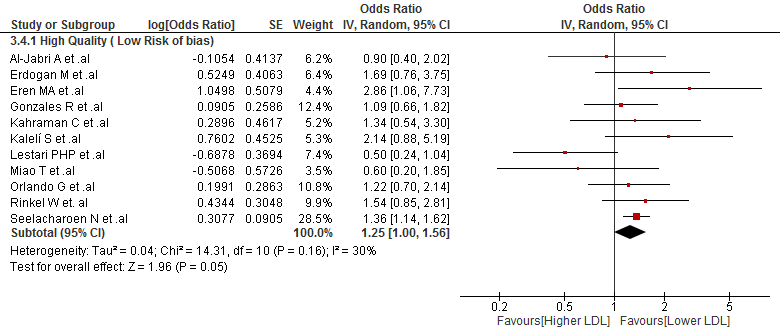
**

**Figure S7. Subgroup analysis according to countries of the association between TG and risk of DFU**

**
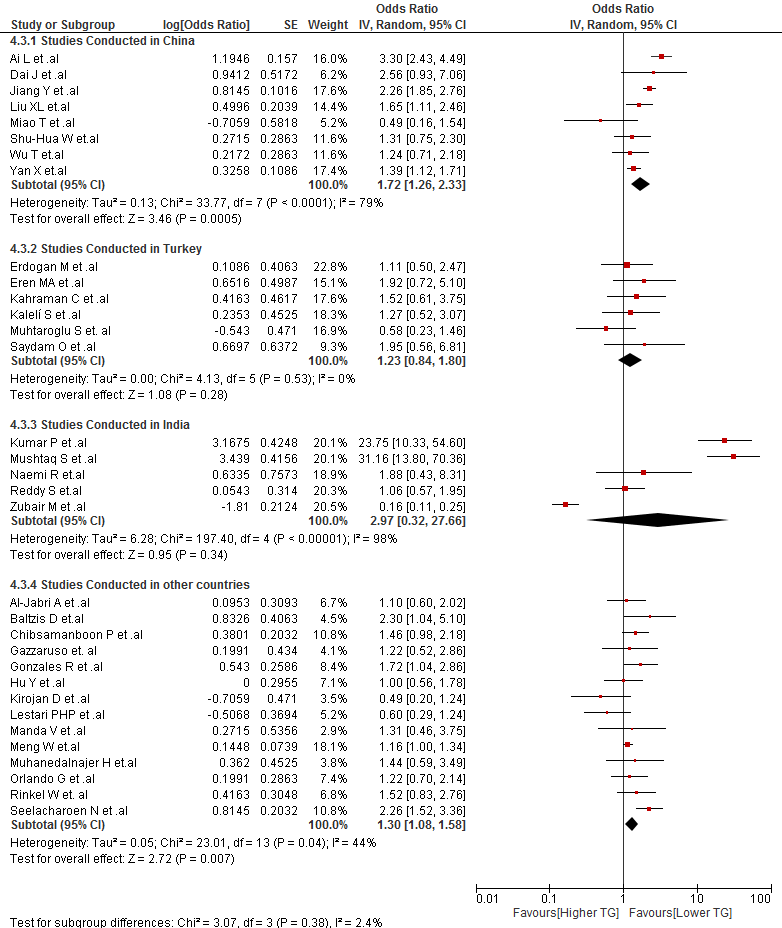
**

**Figure S8. Sensitivity analysis according to risk of bias of the association between TG and risk of DFU**

**
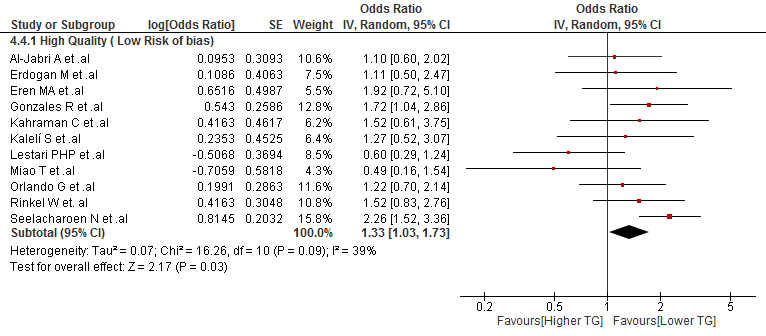
**

**Figure S9. Association of Lp(a) and risk of DFU**


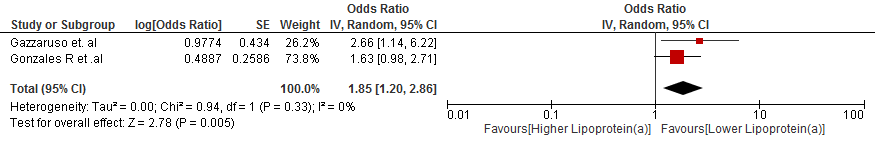


**Figure S10. Association of ApoB and risk of DFU**

**
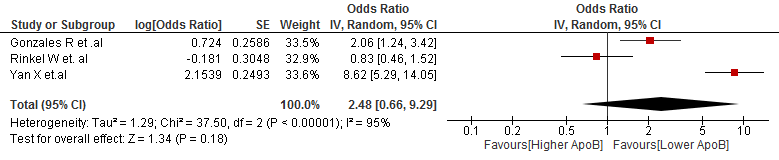
**

**Figure S11. Association of ApoA1 and risk of DFU**

**
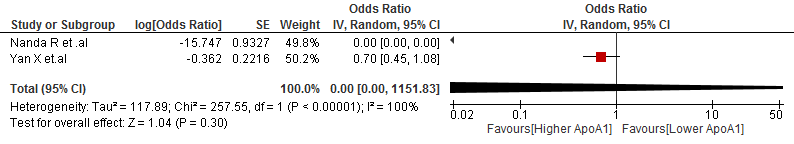
**

**Figure S12. Association of ApoB/ApoA1 ratio and risk of DFU**

**
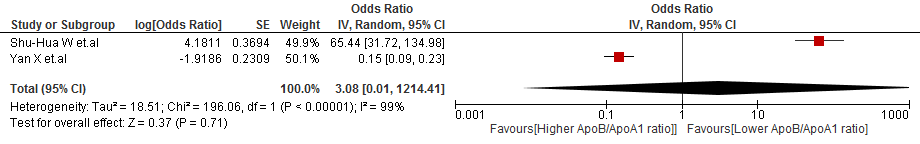
**

**Figure S13. Association of VLDL and risk of DFU**

**
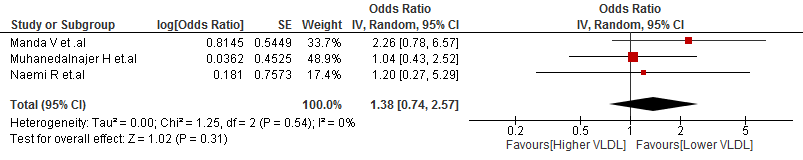
**

**Supplementary Figures S14. Publication bias analysis for all studies that evaluated the association between TC and risk of DFU**

**Figure S14.A Funnel Plot of the studies that evaluated the association between TC and risk of DFU**


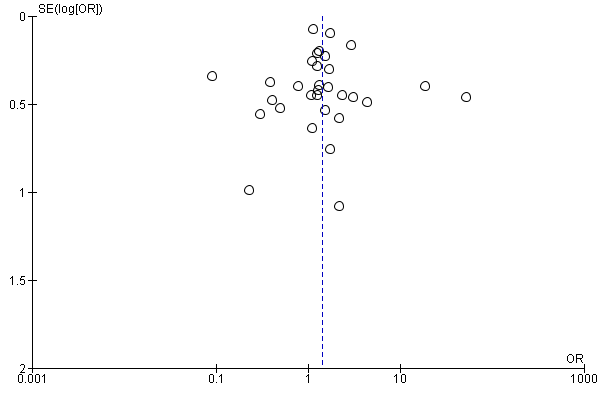


**Figure S14.B Egger Test of all the studies that evaluated the association between TC and risk of DFU**


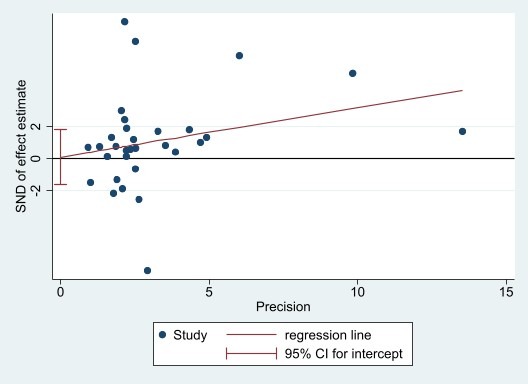


**Supplementary Figures S15. Publication bias analysis for all studies that evaluated the association between HDL and risk of DFU**

**Figure S15.A Funnel Plot of the studies that evaluated the association between HDL and risk of DFU**


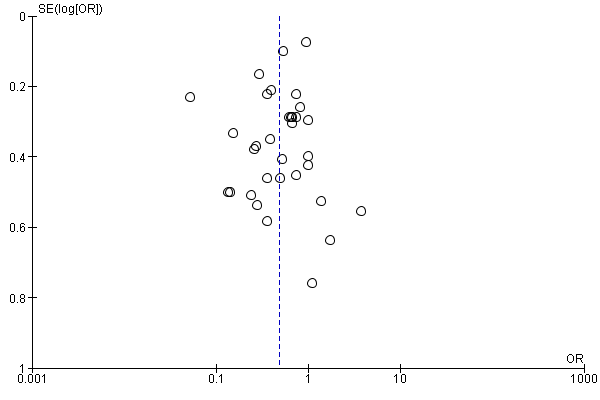


**Figure S15.B Egger Test of all the studies that evaluated the association between HDL and risk of DFU**

**
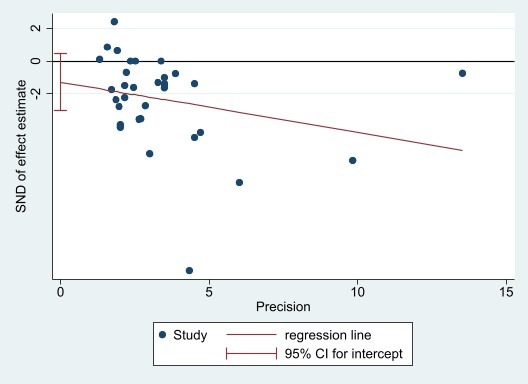
**

**Supplementary Figures S16. Publication bias analysis for all studies that evaluated the association between LDL and risk of DFU**

**Figure S16.A Funnel Plot of the studies that evaluated the association between LDL and risk of DFU**


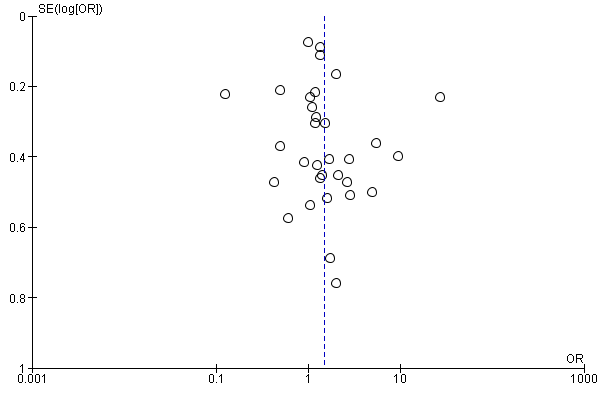


**Figure S16.B Egger Test of all the studies that evaluated the association between LDL and risk of DFU**


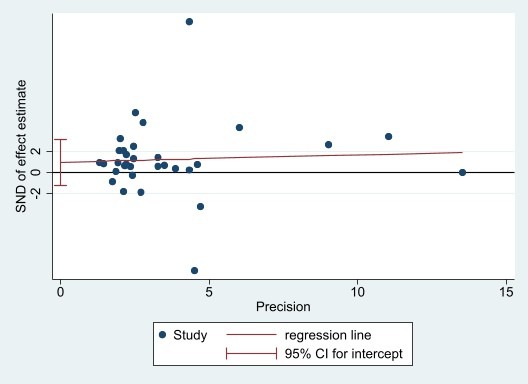


**Supplementary Figures S17. Publication bias analysis for all studies that evaluated the association between TG and risk of DFU**

**Figure S17.A Funnel Plot of the studies that evaluated the association between TG and risk of DFU**


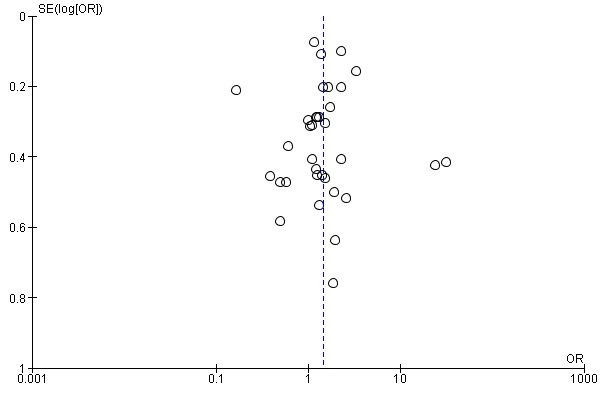


**Figure S17.B Egger Test of all the studies that evaluated the association between TG and risk of DFU**


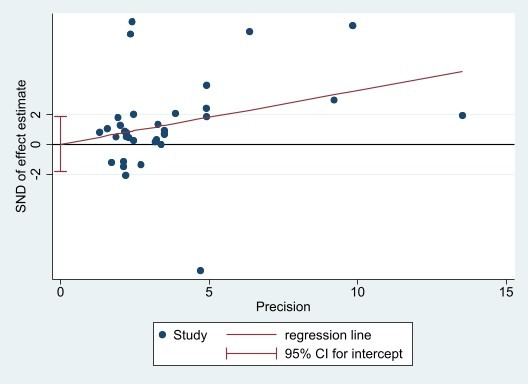

Supplement: Supplementary Materials — Appendix 1: Search strategy. Table S1. PRISMA checklist. Table S2. Newcastle-Ottawa Quality Assessment scale for included studies. Figure S1. Subgroup analysis according to the countries of the association between TC and risk of DFU. Figure S2. Sensitivity analysis according to the risk of bias of the association between TC and risk of DFU. Figure S3. Subgroup analysis according to countries of the association between HDL and risk of DFU. Figure S4. Sensitivity analysis according to the risk of bias of the association between HDL and risk of DFU. Figure S5. Subgroup analysis according to countries of the association between LDL and risk of DFU. Figure S6. Sensitivity analysis according to the risk of bias of the association between LDL and risk of DFU. Figure S7. Subgroup analysis according to countries of the association between TG and risk of DFU. Figure S8. Sensitivity analysis according to the risk of bias of the association between TG and risk of DFU. Figure S9. Association between Lp(a) and risk of DFU. Figure S10. Association between ApoB and risk of DFU. Figure S11. Association between ApoA1 and risk of DFU. Figure S12. Association between ApoB/ApoA1 ratio and risk of DFU. Figure S13. Association between VLDL and risk of DFU. Figure S14. A funnel plot of the studies that evaluated the association between TC and risk of DFU. Figure S14. B Egger test of all the studies that evaluated the association between TC and risk of DFU. Figure S15. A Funnel plot of the studies that evaluated the association between HDL and risk of DFU. Figure S15. B Egger test of all the studies that evaluated the association between HDL and risk of DFU. Figure S16. A Funnel plot of the studies that evaluated the association between LDL and risk of DFU. Figure S16. B Egger test of all the studies that evaluated the association between LDL and risk of DFU. Figure S17. A Funnel plot of the studies that evaluated the association between TG and risk of DFU. Figure S17. B Egger test of all t [file 5450173.f1.docx]
